# The correlation between different antihypertensive treatments and prognosis of cardiovascular disease in hypertensive patients

**DOI:** 10.1186/s12872-023-03381-x

**Published:** 2023-07-22

**Authors:** Shengnan Liu, Fei Li, Chao Zhang, Baozhu Wei, Jing Wan, Hua Shao

**Affiliations:** 1 Department of Cardiovascular Medicine, Zhongnan Hospital of Wuhan University, Shuiguohu Street, Wuchang District, Wuhan City, 430071 Hubei Province China; 2 Department of Electrocardiogram, Zhongnan Hospital of Wuhan University, Wuhan City, China; 3 Renmin Hospital of Wuhan University, Zhangzhidong Road, Wuchang District, Wuhan City, 430071 Hubei Province China

**Keywords:** Hypertension, Antihypertensive treatment, Standard blood pressure control, Adverse cardiovascular events

## Abstract

**Objective:**

To determine the association between different antihypertensive regimens and cardiovascular disease (CVD) outcomes in hypertensive patients.

**Method:**

This single center retrospective cohort study analyzed 602 hypertensive patients with complete medical records at Zhongnan Hospital of Wuhan University, China, from January 2016 to November 2022. Baseline data and follow-up data of the included patients were collected, including demographic and clinical characteristics and laboratory results.

**Results:**

During the 5-year follow-up period, CVD outcomes occurred in 244 hypertensive patients (40.53%). Compared with patients receiving regular antihypertensive treatment, the incidence of adverse cardiovascular events in patients receiving irregular antihypertensive treatment was significantly higher (62 [55.86%] vs 182 [37.07%], HR 1.642, 95% CI 1.227–2.197, *p* < 0.001). In subgroup analysis, the results showed that the incidence of CVD was not identical (χ2 = 9.170,* p* = 0.010). The incidence of adverse cardiovascular events was highest in the single-drug antihypertensive treatment group (43.60%), followed by the multi-drug combination group (41.51%), and lowest in the two-drug combination group (29.58%). Kaplan–Meier curve showed that hypertensive patients treated with two-drug combination antihypertensive had longer overall survival time. We further compared the incidence of CVD between standard blood pressure and intensive blood pressure control, and found no significant difference in the incidence of adverse cardiovascular events between treatment to a systolic blood pressure (SBP) target of less than 140 mmHg compared with a SBP target of less than 120 mmHg (105 [43.93%] vs 35 [29.66%], HR 1.334, 95% CI 0.908–1.961, *p* = 0.142).

**Conclusion:**

The incidence of adverse cardiovascular events was significantly different among different antihypertension treatments. Kaplan–Meier survival curve showed that hypertensive patients receiving two-drug combination antihypertensive treatment had longer overall survival time.

**Supplementary Information:**

The online version contains supplementary material available at 10.1186/s12872-023-03381-x.

## Introduction

Hypertension is a prevalent risk factor for all‐cause morbidity and mortality worldwide, and is also an important modifiable risk factor for preventable global cardiovascular disease (CVD) [1, 2]. Clinical pharmacological treatment with any commonly-used regimen reduces the risk of major cardiovascular events and all-cause mortality, and the benefits of reducing blood pressure on the risks of major CVD are well established [3].

However, the optimal antihypertensive regimen and blood pressure target for hypertensive patients remain controversial. Guideline for the pharmacological treatment of hypertension in adults [4] and the 2017 AHA/ACC blood pressure treatment guideline [5] and the 2018 ESC/ESH guidelines for the management of arterial hypertension [6] recommend that one drug or a combination of two drugs as the initial treatment for hypertension among the vast majority of adults taking antihypertensive medication.

Reliable information about the benefits of different blood-pressure-lowering regimens in preventing CVD events and mortality will be essential for clinical decision-making. A study from the United Kingdom compared the incidence of early cardiovascular events during antihypertensive monotherapy and initial two-drug fixed-dose combination (FDC) shows that this scores in favor of a two-drug strategy as the initial treatment in hypertensive population [7]. Recently, a retrospective study of participants in hypertension clinic have shown that most hypertensive patients require two or more drugs to achieve optimal blood pressure target, and combination therapy had better control efficacy than monotherapy, but previous evidence indicated that treatment adherence is inversely related to the number of drugs taken [8, 9]. Nevertheless, the review published for the first time in 2017 and its second update in 2020 suggests that there was not enough information to draw any conclusion on the relative efficacy of using one medicine versus using two medicines as the initial treatment for hypertensive patients [10, 11].

The relationship between hypertension and major CVD outcomes has always been an important concern of clinicians. It is worth noting that there is too little evidence regarding clinically relevant outcomes under different treatment regimens to draw convincing conclusions about the optimal antihypertensive regimen. To estimate the association between blood-pressure-lowering treatment and risk of major CVD events, we conducted a real-world cohort study to accurately reflect the relationship between different blood-pressure-lowering regimens and CVD outcomes.

## Methods

### Participants

The inclusion criteria for patients in this single-center, retrospective cohort study were: (a) the patients with hypertension admitted to Zhongnan Hospital of Wuhan University, China, from January 2016 to November 2022, (b) all patients met the international diagnostic criteria of hypertension, (c) all patients were followed up regularly and total follow-up time more than 6 months, and (d) the medical records were complete in all patients. Patients were excluded from the study cohort if they met the following criteria: (a) patients with previous adverse cardiovascular events at the time of first hospital diagnosis of hypertension, (b) patients with cerebrovascular disease, respiratory failure, liver and kidney failure, hematologic diseases, malignancy, and active infectious diseases, (c) patients with unscheduled outpatient follow-up or total follow-up time less than 6 months, and (d) hypertensive patients with two or more missing data indicators in medical records. Finally, we included 602 hypertensive patients.

### Study design

In this article, irregular antihypertensive treatment was defined as a treatment regimen with frequent modification of antihypertensive medication or potential cross-over between groups during follow-up period, for example, ACEis/ARBs combined with Beta-blocker antihypertensive treatment regimen was adjusted to ACEis/ARBs + CCBs. While regular antihypertensive treatment was defined as a regimen with no change in the type of antihypertensive medication during long-term follow-up period. In addition, patients with poor compliance or self-discontinuation of antihypertensive treatment were also included in the irregular antihypertensive treatment group.

The primary CVD outcome was a composite of non-fatal myocardial infarction, acute coronary syndrome, malignant arrhythmia, stroke, acute decompensated heart failure, revascularization, and death from cardiovascular causes.

### Data collection

Taking the electronic medical record system of Zhongnan Hospital of Wuhan University as the data source, baseline data and follow-up data, including demographics, physical examination, medical history, comorbidities, laboratory examinations, imaging examinations, therapeutic agents and clinical outcomes, were collected from medical records and laboratory test results registered in the hospital management system, and analyzed at baseline and at the end of follow-up in each treatment group. Blood pressure measurements were taken by a trained nurse. The data were carefully reviewed by 2 independent researchers. Discrepancies between reviewers were resolved by discussion or a third investigator.

### Statistical analysis

Results were summarized using standard statistical evaluations. All statistical analyses were performed with SPSS software (version 24.0; IBM, Armonk, NY) and RStudio (version 2021.09.1.0). All tests were two-tailed and *p* values less than 5% were considered statistically significant. Categorical variables were presented as frequencies and percentages (%), and chi-squared tests were used to compare the proportions for categorical variables. When observed data were limited, the Fisher’s exact tests were used. Continuous variables were expressed as mean ± standard deviation or median (interquartile range [IQR]). When the data were normally distributed, the independent group *t*-tests were used to compare the means for continuous variables, otherwise, the Mann–Whitney* U* tests were used. To test for significant differences between groups, Kruskal–Wallis tests and analysis of variance (ANOVA) tests were used where appropriate. The Cox proportional hazards models were used to analyze differences in event-free survival between patients in different treatment groups. Survival data were visualized by Kaplan–Meier curves and compared using the log-rank tests. Exploratory secondary analyses were performed to examine the treatment effect by intensive and standard blood pressure control.

## Results

### Demographic and baseline characteristics

A total of 602 hypertensive patients were included in this retrospective study. The median age was 67 years (interquartile range 56–77), ranging from 19 to102 years, and 310 patients (51.50%) were female (Appendix Table 1). Total follow-up was 13,387 patient-months (1100 patient-years), and the median follow-up duration of 19 (10–31) months. Among 491 hypertensive patients receiving regular antihypertensive treatment, 172 patients (35%) were treated with single-drug to control blood pressure, 213 patients (43%) were combined with two drugs to reduce blood pressure (91 cases of ACEis/ARBs combined with CCBs, 51 cases of beta-blockers combined with CCBs, 32 cases of ACEis/ARBs combined with beta-blockers, 32 cases of ACEis/ARBs combined with diuretics), and only 106 patients (22%) used more than two drugs to lower blood pressure (Table 1).

**Table 1:** Baseline characteristics of hypertension patients with respect to different antihypertensive treatments

Variables	Normal Range	Regular (*n* = 491)	Single-drug (*n* = 172)	Two-drug (*n* = 213)	Multi-drug (*n* = 106)	*P* value
Age, years	NA	67.00 [56.00, 76.00]	69.00 [58.00, 79.00]	66.00 [57.00, 74.00]	64.50 [51.50, 75.00]	*0.029*
Time, day		574.14 [286.50, 938.50]	626.50 [310.02, 956.20]	578.00 [295.00, 967.00]	556.18 [252.34, 907.00]	0.599
Gender, n (%)	Female	258 (52.55)	105 (61.05)	105 (49.30)	48 (45.28)	*0.017*
	Male	233 (47.45)	67 (38.95)	108 (50.70)	58 (54.72)	
Systolic blood pressure, mmHg	90–139	139.00 [128.00, 152.00]	138.00 [127.00, 150.00]	138.00 [128.00, 151.00]	143.00 [130.00, 159.00]	0.206
Diastolic blood pressure, mmHg	60–89	79.00 [71.00, 88.50]	78.00 [70.00, 86.00]	79.00 [72.00, 88.00]	80.00 [70.00, 93.00]	0.326
Heart rate, bpm	60–100	72.00 [68.00, 78.00]	72.00 [70.00, 78.00]	72.00 [68.00, 78.00]	72.00 [68.00, 80.50]	0.962
Diabetes, n (%)	Yes	110 (22.40)	36 (20.93)	50 (23.47)	24 (22.64)	0.836
	No	381 (77.60)	136 (79.07)	163 (76.53)	82 (77.36)	
Hyperlipidemia, n (%)	Yes	107 (21.79)	38 (22.09)	42 (19.72)	27 (25.47)	0.499
	No	384 (78.21)	134 (77.91)	171 (80.28)	79 (74.53)	
**Laboratory Findings**						
Blood glucose, mmol/L	3.9–6.1	5.29 [4.82, 6.28]	5.26 [4.85, 6.15]	5.30 [4.82, 6.44]	5.28 [4.74, 6.27]	0.808
Serum creatinine, μmol/L	49–90	72.90 [61.20, 87.57]	70.30 [56.00, 84.60]	72.95 [62.92, 85.73]	74.00 [65.65, 93.15]	*0.006*
Blood urea nitrogen, mmol/L	2.8–7.6	5.47 [4.47, 6.60]	5.36 [4.35, 6.48]	5.43 [4.40, 6.58]	5.50 [4.90, 6.88]	0.168
Uric acid, μmol/L	155–357	353.00 [284.88, 437.63]	330.80 [266.22, 398.50]	356.40 [288.90, 453.40]	387.10 [321.80, 465.70]	< *0.001*
Serum calcium ion, mmol/L	2.11–2.52	2.27 [2.18, 2.35]	2.28 [2.21, 2.35]	2.26 [2.17, 2.34]	2.26 [2.18, 2.35]	0.406
Serum potassium ion, mmol/L	3.5–5.3	3.90 [3.65, 4.16]	3.94 [3.74, 4.17]	3.88 [3.66, 4.16]	3.86 [3.57, 4.10]	0.101
Serum sodium ion, mmol/L	137–147	140.50 [138.60, 142.10]	140.25 [138.50, 141.93]	140.65 [138.57, 142.33]	140.65 [139.28, 142.15]	0.476
Total cholesterol, mmol/L	< 5.18	4.49 [3.70, 5.12]	4.57 [3.78, 5.21]	4.61 [3.77, 5.18]	4.07 [3.53, 4.98]	*0.016*
High density lipoprotein, mmol/L	> 1.04	1.14 [0.95, 1.36]	1.21 [0.98, 1.41]	1.14 [0.94, 1.32]	1.08 [0.89, 1.26]	*0.012*
Low density lipoprotein, mmol/L	< 3.37	2.67 [2.12, 3.22]	2.70 [2.18, 3.22]	2.73 [2.20, 3.26]	2.38 [1.98, 3.01]	*0.036*
Triglyceride, mmol/L	< 1.7	1.43 [1.03, 2.04]	1.35 [0.95, 1.94]	1.49 [1.06, 2.16]	1.42 [1.10, 2.03]	0.153
Creatine kinase, U/L	< 145	88.00 [66.00, 126.75]	88.50 [69.00, 130.25]	87.50 [65.75, 122.25]	86.00 [65.25, 135.75]	0.728
Creatine kinase-MB, U/L	0–25	12.00 [9.00, 17.00]	12.00 [9.00, 18.00]	12.00 [8.00, 16.00]	12.00 [9.00, 17.00]	0.495
Lactate dehydrogenase, U/L	125–243	190.00 [166.00, 223.50]	189.00 [168.75, 220.00]	187.00 [166.00, 223.00]	194.50 [162.00, 227.50]	0.928
Cardiac troponin I, pg/mL	0–26.2	4.00 [2.00, 9.90]	3.40 [1.90, 8.90]	4.00 [2.20, 8.40]	4.90 [2.10, 12.40]	0.218
N-terminal pro-brain natriuretic peptide, pg/mL	< 100	113.00 [57.00, 345.00]	97.75 [52.65, 291.50]	112.00 [66.60, 346.00]	188.50 [69.55, 552.75]	0.121
**Echocardiography**						
Ascending aorta diameter, mm	20–34	33.00 [29.08, 35.00]	32.00 [30.00, 35.00]	32.50 [29.00, 35.00]	34.00 [31.00, 35.50]	0.090
Left atrial diameter, mm	22–36	34.00 [31.00, 37.00]	33.00 [32.00, 36.00]	34.00 [30.00, 37.00]	35.00 [32.75, 38.00]	0.101
Left ventricular diameter, mm	36–53	44.00 [41.00, 47.00]	44.00 [41.00, 46.00]	44.00 [41.00, 48.00]	45.00 [43.00, 48.00]	*0.020*
Ventricular septal thickness, mm	6–11	11.00 [10.00, 12.00]	11.00 [10.00, 12.00]	11.00 [10.00, 12.00]	11.00 [10.00, 12.00]	0.112
Pulmonary artery diameter, mm	14–26	24.00 [23.00, 26.00]	24.00 [23.00, 25.00]	24.00 [23.00, 26.00]	24.00 [23.00, 26.00]	0.319
LVEF, (%)	50–75	67.00 [61.00, 71.00]	67.00 [64.00, 71.25]	65.00 [60.00, 70.25]	67.00 [60.75, 70.25]	*0.026*
Severe valve regurgitation, n (%)	Yes	84 (17.11)	28 (16.28)	37 (17.37)	19 (17.92)	0.931
	No	407 (82.89)	144 (83.72)	176 (82.63)	87 (82.08)	
Severe valve calcification, n (%)	Yes	73 (14.87)	28 (16.28)	25 (11.74)	20 (18.87)	0.196
	No	418 (85.13)	144 (83.72)	188 (88.26)	86 (81.13)	

*NA* Not available. *P* values < 0.05 are written in italics

Values shown are mean ± SD, median (interquartile range [IQR]) or n (%). *P* values were calculated by chi-squared test, Fisher’s exact test, t test, or Mann–Whitney U test, as appropriate

*Abbreviations*: *n* Number, *LVEF* Left ventricular ejection fraction

The level of serum calcium ion was significantly higher, while the levels of total cholesterol, pulmonary artery diameter were significantly lower in patients receiving regular antihypertensive treatment (*p* < 0.05). In addition, the proportion of hypertensive patients with regular antihypertensive treatment combined with hyperlipidemia was significantly higher than that of patients with irregular antihypertensive treatment (107[21.79%] vs 14[12.61%], *p* = 0.041). In other aspects, there were no significant differences in baseline characteristics of hypertensive patients between regular and irregular antihypertensive groups (*p* > 0.05 for all) (Appendix Table 1).

In each antihypertensive treatment group, there was no difference in blood pressure, heart rate, blood glucose, blood urea nitrogen, ion level, myocardial enzymes, and pro-brain natriuretic peptide levels in hypertensive patients (*p* > 0.05 for all). Notably, patients in different antihypertensive treatment groups differed with multiple indexes of blood lipids and organ function, including the serum creatinine (*p* = 0.006), blood uric acid (*p* < 0.001), total cholesterol (*p* = 0.016), high density lipoprotein (*p* = 0.012), low density lipoprotein (*p* = 0.036). Compared with hypertensive patients in the two-drug and multi-drug antihypertensive treatment groups, the age (*p* = 0.029) and the proportion of female (*p* = 0.017) was significantly higher in the single-drug treatment group. Furthermore, in patients receiving two-drug treatment regimen, the levels of total cholesterol (*p* = 0.016), low density lipoprotein (*p* = 0.036) were significantly higher than in patients receiving single-drug and multi-drug treatment. The results of echocardiographic showed that left ventricular diameter which associated with cardiac remodeling was highest in hypertensive patients receiving multi-drug combination antihypertensive treatment, followed by the two-drug combination treatment group and single-drug antihypertensive treatment group (Table 1).

### Follow-up characteristics

At follow-up, the proportion of hypertensive patients with regular antihypertensive treatment combined with diabetes was significantly higher than that of patients with irregular antihypertensive treatment.(138[28.11] vs 20[18.02], *p* = 0.039) (Appendix Table 2).

Hypertensive patients receiving multi-drug antihypertensive treatment had significantly higher serum creatinine, blood uric acid, triglyceride, and left ventricular diameter levels and lower total cholesterol level (*p* < 0.05) (Appendix Table 3).

### Cardiovascular disease outcomes

The data of clinical outcomes are summarized in Table 2. Among 602 patients, 244 hypertensive patients (40.53%) developed CVD outcomes. Hypertensive patients receiving irregular pharmacological antihypertensive treatment were more likely to exhibit adverse cardiovascular events compared with patients receiving regular antihypertensive treatment (62 [55.86%] vs 182 [37.07%], χ2 = 12.491, *p* < 0.001).

**Table 2:** Cardiovascular disease outcomes of hypertension patients with different antihypertensive treatments

Antihypertensive treatment	Hypertension (n)	Cardiovascular disease (n)	Percentage of cardiovascular disease outcomes (%)	${\mathrm{x}}^{2}\mathrm{value}$	*P* value
Regular	309	182	37.07	12.491	< *0.001*
Irregular	49	62	55.86		
Single-drug	97	75	43.60	9.170	*0.010*
Two-drug	150	63	29.58		
Multi-drug	62	44	41.51		
Single-drug	97	75	43.60	7.544	*0.006*
Two-drug	150	63	29.58		
Two-drug	150	63	29.58	4.001	*0.045*
Multi-drug	62	44	41.51		
Single-drug	97	75	43.60	0.048	0.827
Multi-drug	62	44	41.51		
ACEis/ARBs + Beta-blockers	20	12	37.5	8.853	*0.031*
ACEis/ARBs + CCBs	73	18	19.8		
ACEis/ARBs + Diuretics	24	8	25.0		
Beta-blockers + CCBs	30	21	41.1		

*P* values < 0.05 are written in italics

Values shown are n or (%). *P* values were calculated by chi-squared test, or Fisher’s exact test, as appropriate

*Abbreviations*: *n* Number, *ACEi* Angiotensin-converting enzyme inhibitor, *ARB* Angiotensin receptor antagonist, *CCB* Calcium channel receptor blocker

Of the 491 patients receiving antihypertensive treatment patients, 182 hypertensive patients (37.07%) developed CVD outcomes, 43.60% (75 of 172) received single-drug treatment, 41.51% (44 of 106) received multi-drug combination antihypertensive treatment, and 29.58% (63 of 213) received two-drug combination antihypertensive treatment. Hypertensive patients in different antihypertensive treatment groups significantly differed with the incidence of CVD outcomes (single-drug vs two-drug vs multi-drug, 75 [43.60%] vs 63 [29.58%] vs 44 [41.51%], χ2 = 9.170, *p* = 0.010).

Compared with hypertensive patients in the two-drug antihypertensive treatment group, the incidences of CVD were significantly higher in the single-drug and multi-drug treatment groups (75 [43.60%] vs 63 [29.58%], χ2 = 7.544, *p* = 0.006, and 44 [41.51%] vs 63 [29.58%], χ2 = 4.001, *p* = 0.045, respectively). However, there was no significant differences in the incidences of adverse cardiovascular events between the single-drug and the multi-drug treatment group (75 [43.60%] vs 44 [41.51%], χ2 = 0.048, *p* = 0.827).

**Fig. 1:**
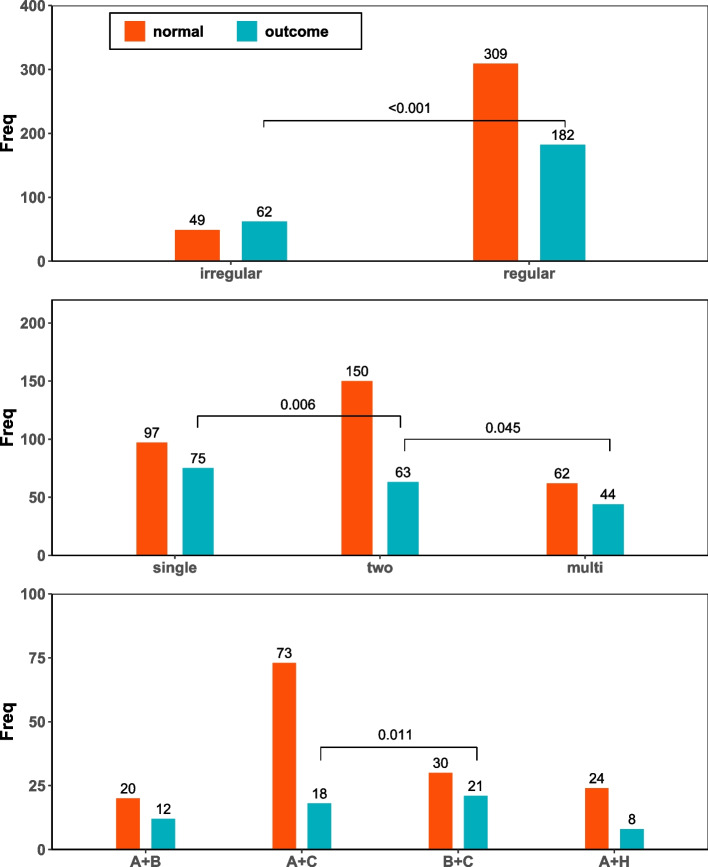
Cardiovascular disease outcomes of hypertension patients with different antihypertensive treatments

Exploratory analysis of hypertensive patients treated with two-drug antihypertensive treatment indicated that there were significant differences in the incidence of adverse cardiovascular events among the four treatment subgroups (ACEis/ARBs + Beta-blocker vs ACEis/ARBs + CCBs vs ACEis/ARBs + Diuretics vs Beta-blockers + CCBs, 12 [37.5%] vs 18 [19.8%] vs 8 [25.0%] vs 21 [41.1%], χ2 = 8.853, *p* = 0.031). In addition, hypertensive patients treated with the A + C regimen had a significantly lower incidence of cardiovascular events than patients treated with the B + C regimen (*p* = 0.011) (Fig. 1).

### Survival curve analysis

In this study, Kaplan–Meier method and log-rank test were used to investigate the relationship between antihypertensive treatment and prognosis of hypertension. The results indicated that the overall event-free survival of hypertensive patients treated with regular antihypertensive treatment was significantly higher than that of patients treated with irregular antihypertensive treatment (HR 1.642, 95% CI 1.227–2.197, *p* < 0.001) (Appendix Fig. 1).

**Fig. 2:**
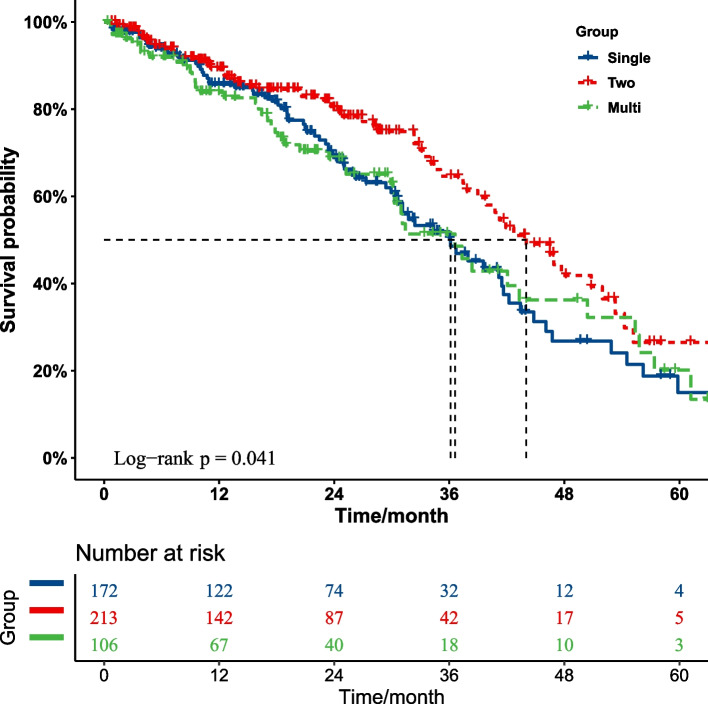
Kaplan–Meier curves of event-free survival for adverse cardiovascular events over 5 years in hypertensive patients receiving different antihypertensive treatment

Kaplan–Meier survival curves for hypertensive patients receiving different antihypertensive treatment are presented in Fig. 2 and Appendix Fig. 2. Event-free survival for adverse cardiovascular events in hypertensive patients differed significantly between different antihypertensive treatments (single-drug vs two-drug vs multi-drug, *p* = 0.041).

Overall event-free survival was significantly lower in the two-drug antihypertensive treatment group compared with hypertensive patients in the single-drug antihypertensive treatment group (HR 0.651, 95% CI 0.464–0.913, *p* = 0.012). However, there was no statistically significant difference in cardiovascular event-free survival in hypertensive patients between the multi-drug and other treatment groups (*p* > 0.05 for all).

In addition, subgroup analysis of hypertensive patients receiving two-drug antihypertensive treatment showed no significant difference in event-free survival for adverse cardiovascular events between the four treatment subgroups, indicating that all treatments were non-inferior to any other treatment (ACEis/ARBs + Beta-blocker vs ACEis/ARBs + CCBs vs ACEis/ARBs + Diuretics vs Beta-blockers + CCBs,* p* = 0.53) (Appendix Fig. 3).

**Fig. 3:**
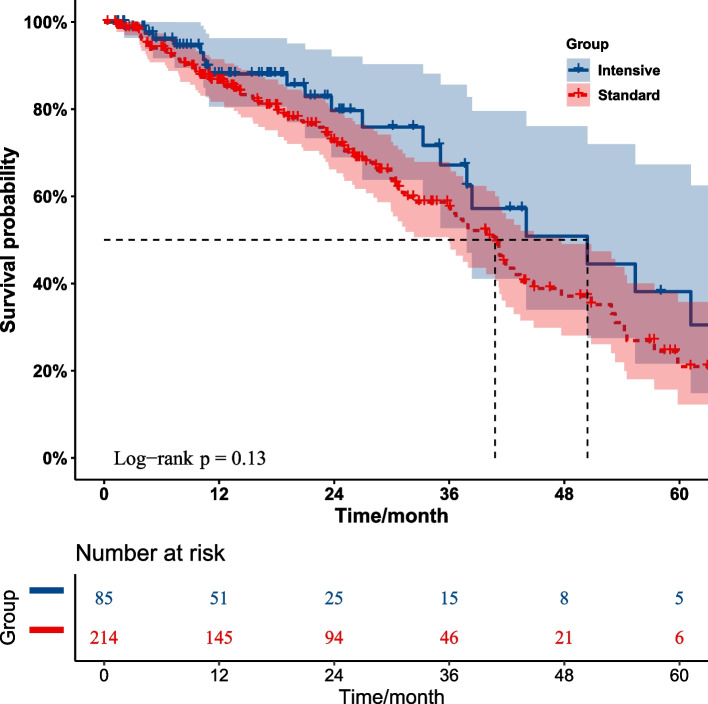
Kaplan–Meier curves of event-free survival for adverse cardiovascular events over 5 years in hypertensive patients on intensive or standard treatment in the regular antihypertensive treatment group

### Intensive vs standard blood pressure control

Among the 491 hypertensive patients receiving regular antihypertensive treatment, 299 patients had blood pressure controlled within the normal range. To evaluate the effects of intensive (less than 120 mmHg) versus standard (less than 140 mmHg) SBP targets in hypertensive patients, we divided hypertensive patients into intensive treatment group (n = 85) and standard treatment group (n = 214) according to their follow-up results of 24-h ambulatory blood pressure monitoring. During the 5-year follow-up period, patients divided into the intensive-treatment arm achieved a mean SBP of 110.85 mmHg, whereas those in the standard-treatment arm achieved a mean SBP of 129.42 mmHg. The incidence of diabetes mellitus and hyperlipidemia did not differ between these treatment groups (24 [28.24%] vs 50 [23.36%], *p* = 0.464, and 22 [25.88%] vs 62 [28.97%], *p* = 0.694, respectively) (Table 3). In the intensive treatment group, patients with hypertension had lower levels of total cholesterol, low density lipoprotein, and pro-brain natriuretic peptide compared with patients in the standard treatment group (3.67 [3.28, 4.27] vs 4.16 [3.32, 5.02] mmol/L,* p* = 0.003, 2.13 [1.66, 2.57] vs 2.42 [1.74, 3.18] mmol/L, *p* = 0.014, and 58.10 [24.47, 261.00] vs 99.70 [59.90, 408.50] mmol/L, *p* = 0.014, respectively). Most characteristics were similar between treatment groups except for lipid profile and pro-brain natriuretic peptide.

**Table 3:** Clinical, laboratory and imaging data of hypertension patients among different antihypertensive targets

Variables	Intensive Treatment (*n* = 85)		Standard Treatment (*n* = 214)		*P* value ^a^
	Pre-treatment	Post-treatment	Pre-treatment	Post-treatment	
Systolic pressure, mmHg	133.00 [123.00, 147.00]	112.00 [106.00, 117.00]	134.00 [126.00, 151.00]	130.00 [125.00, 134.00]	** < *0.001***
Diastolic pressure, mmHg	79.00 [71.00, 87.00]	73.00 [67.00, 80.00]	78.00 [70.00, 86.00]	76.00 [69.25, 83.00]	0.103
Heart rate, bpm	72.00 [66.00, 78.25]	72.00 [66.00, 78.00]	72.00 [69.00, 78.00]	74.00 [68.00, 80.00]	0.398
**Laboratory Findings**					
Venous blood glucose, mmol/L	5.00 [4.69, 5.94]	5.30 [5.02, 5.90]	5.20 [4.83, 6.03]	5.28 [4.93, 6.16]	0.778
Serum creatinine, μmol/L	73.80 [61.05, 86.00]	74.20 [65.47, 92.17]	70.30 [57.35, 86.90]	69.40 [58.20, 90.00]	0.061
Blood urea nitrogen, mmol/L	5.30 [4.51, 6.12]	5.78 [4.57, 7.18]	5.30 [4.45, 6.50]	5.70 [4.60, 7.13]	0.857
Uric acid, μmol/L	353.70 [305.70, 437.00]	379.10 [314.22, 449.78]	346.10 [266.75, 430.40]	351.40 [277.60, 425.10]	***0.011***
Calcium ion, mmol/L	2.24 [2.16, 2.32]	2.25 [2.17, 2.34]	2.27 [2.16, 2.36]	2.25 [2.19, 2.34]	0.801
Potassium ion, mmol/L	3.95 [3.74, 4.12]	3.94 [3.72, 4.15]	3.87 [3.64, 4.16]	3.95 [3.70, 4.14]	0.972
Sodium ion, mmol/L	140.75 [138.70, 142.35]	140.00 [138.00, 142.00]	140.40 [138.60, 141.80]	140.60 [138.80, 142.20]	0.248
Total cholesterol, mmol/L	4.18 [3.68, 4.88]	3.67 [3.28, 4.27]	4.56 [3.71, 5.17]	4.16 [3.32, 5.02]	***0.003***
High density lipoprotein, mmol/L	1.17 [1.01, 1.34]	1.09 [0.92, 1.27]	1.14 [0.94, 1.40]	1.13 [0.93, 1.30]	0.435
Low density lipoprotein, mmol/L	2.50 [2.12, 3.12]	2.13 [1.66, 2.57]	2.70 [2.14, 3.24]	2.42 [1.74, 3.18]	***0.014***
Triglyceride, mmol/L	1.33 [1.05, 1.80]	1.16 [0.84, 1.65]	1.42 [0.99, 2.01]	1.29 [0.92, 1.95]	0.197
Creatine kinase, U/L	81.00 [66.25, 125.62]	96.00 [73.25, 131.25]	89.00 [68.00, 135.25]	94.00 [66.00, 137.00]	0.841
Creatine kinase-MB, U/L	12.00 [10.00, 15.50]	13.00 [10.00, 20.00]	12.00 [8.00, 15.75]	13.00 [10.00, 17.00]	0.347
Lactate dehydrogenase, U/L	190.50 [170.25, 218.75]	190.00 [167.00, 227.75]	189.00 [167.25, 220.00]	186.00 [164.00, 211.00]	0.453
Cardiac troponin I, pg/mL	3.90 [1.90, 9.15]	3.60 [1.90, 12.70]	3.30 [1.90, 8.90]	3.70 [1.90, 10.10]	0.958
Pro-brain natriuretic peptide, pg/mL	79.05 [40.47, 266.00]	58.10 [24.47, 261.00]	111.00 [67.75, 312.25]	99.70 [59.90, 408.50]	***0.014***
**Echocardiography**					
Ascending aorta diameter, mm	33.00 [28.25, 34.00]	32.00 [30.00, 34.00]	32.00 [29.00, 35.00]	33.00 [30.00, 35.00]	0.177
Left atrial diameter, mm	33.00 [30.00, 36.00]	34.00 [31.50, 37.50]	34.00 [31.00, 37.00]	35.00 [31.00, 39.00]	0.324
Left ventricular diameter, mm	43.00 [41.00, 46.00]	45.00 [41.50, 48.00]	44.00 [41.00, 47.25]	44.50 [42.00, 48.00]	0.957
Ventricular septal thickness, mm	11.00 [10.00, 12.00]	11.00 [10.00, 12.00]	11.00 [10.00, 12.00]	11.00 [10.00, 12.00]	0.855
Pulmonary artery diameter, mm	24.00 [23.00, 25.50]	24.00 [22.00, 25.75]	24.00 [23.00, 26.00]	24.00 [21.00, 26.00]	0.881
LVEF, (%)	66.00 [61.50, 71.00]	65.00 [59.00, 69.00]	65.00 [61.00, 70.00]	63.00 [59.00, 68.00]	0.662
Severe valve regurgitation, n (%)	13 (15.29)	26 (30.59)	39 (18.22)	77 (35.98)	0.453
Severe valve calcification, n (%)	12 (14.12)	17 (20.00)	31 (14.49)	46 (21.50)	0.898
**Complications**					
Diabetes, n (%)	15 (17.65)	24 (28.24)	38 (17.76)	50 (23.36)	0.464
Hyperlipidemia, n (%)	13 (15.29)	22 (25.88)	45 (21.03)	62 (28.97)	0.694
**Treatments**					
Single-drug, n (%)		28 (32.94)		84 (39.25)	0.565
Two-drug, n (%)		39 (45.88)		92 (42.99)	
Multi-drug, n (%)		18 (21.18)		38 (17.76)	
**Clinical outcomes**		21 (24.71)		86 (40.19)	***0.017***

*N*A Not available. *P* values < 0.05 are written in italics

Values shown are mean ± SD, median (interquartile range [IQR]) or n (%). *P* values were calculated by chi-squared test, Fisher’s exact test,* t* test, or Mann–Whitney *U* test, as appropriate

*Abbreviations*: *n* Number, *LVEF* Left ventricular ejection fraction

^a^Statistical difference between the intensive group and standard group after treatment

In terms of clinical outcomes, the treatment effect estimates for adverse CVD were statistically significant between treatment groups. Primary cardiovascular outcome events were observed for 21 patients in the intensive treatment group and for 86 patients in the standard treatment group. Within the standard treatment group, the incidence of major cardiovascular events was significantly higher than those in the intensive treatment group (21 [24.71%] vs 86 [40.19%], *p* = 0.017) (Table 3). However, Kaplan–Meier survival curves showed no significant difference in overall event-free survival time between patients with a SBP target of less than 140 mmHg and those with a SBP target of less than 120 mmHg (HR 1.445, 95% CI 0.893–2.338, *p* = 0.134) (Fig. 3). Similarly, in the subgroup analysis for hypertensive patients receiving two-drug antihypertensive treatment, there was no significant difference in event-free survival for adverse cardiovascular events between treatment groups (HR 1.025, 95% CI 0.499–2.104, *p* = 0.946) (Appendix Fig. 4). It is worth noting that, there was a significant difference in event-free survival for adverse cardiovascular events between intensive and standard treatment hypertensive patients in the single-drug treatment group (*p* = 0.046) (Appendix Fig. 5).

## Discussion

As a major global public health problem, the preferential use of superior antihypertensive regimens is imperative to guide hypertensive patients and reduce the global burden of CVD [2]. Based on the analysis of different SBP targets, we combined antihypertensive regimens to analyze the prognosis of CVD and provide the latest evidence to estimate the comparative efficacy of different antihypertensive treatments and SBP targets on the risks of major cardiovascular events in clinical trials.

Our study followed a retrospective cohort design. We analyzed data from 602 patients and provided detailed information on the association between antihypertensive regimens, blood pressure targets, and major CVD outcomes in hypertensive patients. The results indicate that hypertensive patients treated with irregular antihypertensive treatment were more likely to develop adverse cardiovascular events compared with patients treated with regular antihypertensive treatment. The results presented herein are consistent with those from previous observational studies [12–15] and a network meta-analysis [16] of clinical trials, showing that major first-line antihypertension medications, including ACE inhibitors, ARBs, β-blockers, DH CCBs, and diuretics, were all reported to be effective in reducing cardiovascular events.

Among 491 patients receiving antihypertensive treatment, first, we compared differences in demographic and clinical characteristics between the three antihypertensive treatment groups. These data showed that lipid profile level was significantly higher in hypertensive patients treated with the combination of two drugs, indicating that clinicians were more likely to prescribe two drugs combined with hypertension when patients with elevated lipid profile. Clinical outcomes suggested that the incidence of adverse cardiovascular events was significantly higher in the single-drug antihypertensive treatment group than in the two-drug treatment group. The incidence of the primary clinical outcomes was higher than expected, which may be attributed to the lower proportion of SBP achieving an intensive-treatment target in the single-drug treatment group (32.94%), and does not exclude the influence of multiple potential confounders. Second, Kaplan–Meier survival analysis showed that overall survival time was longer in patients treated with two-drug combination antihypertensive treatment. Our findings further supported this evidence that the combination of two antihypertension medications was more effective, with fewer adverse cardiovascular events, in reducing blood pressure than standard monotherapy [7, 17]. Furthermore, we found no evidence of significant differences in event-free survival for adverse cardiovascular events among hypertensive patients prescribed ACEis/ARBs combined with Beta-blocker, ACEis/ARBs combined with CCBs, ACEis/ARBs + diuretics, or Beta-blockers combined with CCBs, which were associated with similar benefits in reducing cardiovascular events, probably due to the limited sample size in this study. Future studies should compare the effectiveness of more combinations in reducing cardiovascular events, such as β-blockers and diuretics.

Recent analyses comparing different blood pressure targets have found that more intensive treatment was beneficial for residual life span and potential survival gains compared to less intensive treatment [18]. In order to determine the effect of blood pressure target on the prognosis of CVD in hypertensive patients, Kaplan–Meier survival curves were applied in this study, and the results supported the evidence indicating that treating to a SBP target of less than 140 mmHg resulted in no significant difference in overall event-free survival compared with a SBP target of less than 120 mmHg.

However, under different antihypertensive regimens, what is the association between blood pressure targets and CVD outcomes? Hypertensive patients were divided into single-drug and two-drug combination treatment groups, and according to different blood-pressure-lowering regimens, we further analyzed the relative efficacy of standard blood pressure versus intensive blood pressure control. We found that Kaplan Meier survival curve for the single-drug treatment group showed that hypertensive patients with a SBP target of less than 120 mmHg had longer event-free survival time. In contrast to intention-to-treat analysis, we focused our study on blood pressure targets and antihypertensive regimens at follow-up. Therefore, our results should be interpreted as a desirable blood pressure to achieve in maintenance therapy, which was consistent with previous randomized clinical trials and meta-analyses, indicating that intensive antihypertensive regimen to lower blood pressure targets reduces the risk for CVD and mortality [19–22].

In contrast, in the two-drug combined treatment group, no survival advantage benefit of intensive blood pressure control was observed at all. Moreover, in terms of the impact on various clinical characteristics under the targets of reducing blood pressure, the uric acid level of hypertensive patients in the intensive treatment group was higher and the lipid profile levels were lower than those in the standard treatment group, which indicates that the intensive treatment group may damage renal function of patients to a certain extent, but lower lipid profile levels.

Our study has several limitations. First, this study was based on a single-center study, we completely excluded patients with potential cross-over between different medications during follow-up period, which leads to the small sample size being a major limitation of the present study, and given the prevalence of the investigated diseases, more large-scale multicenter studies were needed to validate our conclusions. Second, as a retrospective cohort study, relevant data on cardiovascular complications, treatment drugs for diabetic patients and some other specific information on echocardiography in this study are missing due to the lack of clinical data, so there may be a return visit bias, especially the overall confounding factors such as the treatment drugs and compliance of diabetic patients may have some influence on our research results, which requires further research. Here, it was important to note that our results merely indicate that treatment to a SBP target of less than 120 mmHg was superior to a SBP target of less than 140 mmHg in patients at high risk for cardiovascular events receiving single-drug antihypertensive treatment. However, the lower incidence of primary outcome events in the intensive treatment group with lower diastolic blood pressure (DBP) in SPRINT (Systolic Pressure Intervention Trial) was associated with increased clinically significant serious adverse events (SAEs) [19]. An important limitation of our study was the lack of analysis of different DBP targets and subsequent serious adverse events. If lower SBP was adopted as the target, it is important to monitor patients more carefully for adverse effects of treatment [20]. Third, because there were too few partial permutation data for the combination, large clinical trials evaluating and reporting more clinically relevant endpoints were needed. Ideally, future studies should include results from all combinations of antihypertension medications. Given the possibility of residual confounding and the limited length of observation periods, more frequently studies were warranted to determine the potential impact of pharmacological therapies on CVD events among hypertensive patients in the future, and we are looking forward to the results of ongoing randomized clinical trial.

## Conclusion

Different antihypertensive treatments and blood pressure targets affect CVD outcomes in hypertensive patients. Kaplan–Meier survival curve showed that hypertensive patients treated with two-drug combination antihypertensive had a longer overall survival time. When treating to a SBP target of less than 120 mmHg, the incidence of major adverse cardiovascular events was significantly lower in the single-drug antihypertensive treatment group. However, no survival advantage benefit of intensive blood pressure control was observed at all in the two-drug combined treatment group, and this analysis may help clinicians and patients make decisions regarding the intensity and regimens of blood pressure treatment to prevent cardiovascular events and death in hypertensive patients.

## Supplementary Information

**Additional file 1: Appendix Table 1.** Baseline Characteristics of Hypertension Patients with respect to different antihypertensive treatments.

**Additional file 2: Appendix Table 2.** Follow-up Characteristics of Hypertension Patients with respect to different antihypertensive treatments.

**Additional file 3: Appendix Table 3.** Follow-up Characteristics of Hypertension Patients with respect to different antihypertensive treatments.

**Additional file 4: Appendix Figure 1. **Kaplan-Meier curves of event-free survival for adverse cardiovascular events over 5 years in patients receiving regular or irregular antihypertensive treatment. **Appendix Figure 2. **Kaplan-Meier curves of event-free survival for adverse cardiovascular events over 5 years in patients receiving single-drug and two-drug antihypertensive treatment. **Appendix Figure 3. **Kaplan-Meier curves of event-free survival for adverse cardiovascular events over 5 years in patients receiving ACEis/ARBs + Beta-blockers, ACEis/ARBs + CCBs, Beta-blockers + CCBs and ACEis/ARBs +Diuretics, respectively. **Appendix Figure 4.** Kaplan-Meier curves of event-free survival for adverse cardiovascular events over 5 years in hypertensive patients on intensive or standard treatment in the two-drug antihypertensive treatment group. **Appendix Figure 5.** Kaplan-Meier curves of event-free survival for adverse cardiovascular events over 5 years in hypertensive patients on intensive or standard treatment in the single-drug antihypertensive treatment group.

## Data Availability

The datasets used and/or analyzed during the current study available from the corresponding author on reasonable request.
