# Supplementary material for: The correlation between different antihypertensive treatments and prognosis of cardiovascular disease in hypertensive patients
Source: BMC Cardiovasc Disord. 2023 Jul 22;23:369. doi: 10.1186/s12872-023-03381-x (PMC10363321; doi:10.1186/s12872-023-03381-x)
Supplement: Supplementary file 1 — Additional file 1: Appendix Table 1. Baseline Characteristics of Hypertension Patients with respect to different antihypertensive treatments. [file 12872_2023_3381_MOESM1_ESM.docx]

**Appendix Table 1. Baseline Characteristics of Hypertension Patients with respect to different antihypertensive treatments**

| Variables | Normal Range | All patients (n=602) | Irregular (n=111) | Regular (n=491) | *P* value |
| --- | --- | --- | --- | --- | --- |
| Age, years | NA | 67.00 [56.00, 77.00] | 68.00 [53.50, 79.00] | 67.00 [56.00, 76.00] | 0.954 |
| Time, day |  | 574.57 [302.00, 931.25] | 622.00 [355.50, 917.50] | 574.14 [286.50, 938.50] | 0.485 |
| Gender, n (%) | Female | 310 (51.50) | 52 (46.85) | 258 (52.55) | 0.327 |
|  | Male | 292 (48.50) | 59 (53.15) | 233 (47.45) |  |
| Systolic blood pressure, mmHg | 90-139 | 139.00 [128.00, 152.00] | 140.00 [129.50, 152.00] | 139.00 [128.00, 152.00] | 0.938 |
| Diastolic blood pressure, mmHg | 60-89 | 79.00 [71.25, 89.75] | 80.00 [74.50, 91.00] | 79.00 [71.00, 88.50] | 0.114 |
| Heart rate, bpm | 60-100 | 72.00 [69.00, 79.75] | 74.00 [70.00, 81.75] | 72.00 [68.00, 78.00] | 0.125 |
| Diabetes, n (%) | Yes | 129 (21.43) | 19 (17.12) | 110 (22.40) | 0.272 |
|  | No | 473 (78.57) | 92 (82.88) | 381 (77.60) |  |
| Hyperlipidemia, n (%) | Yes | 121 (20.10) | 14 (12.61) | 107 (21.79) | *0.041* |
|  | No | 481 (79.90) | 97 (87.39) | 384 (78.21) |  |
| **Laboratory Findings** |  |  |  |  |  |
| Blood glucose, mmol/L | 3.9-6.1 | 5.28 [4.83, 6.23] | 5.23 [4.93, 5.97] | 5.29 [4.82, 6.28] | 0.969 |
| Serum creatinine, μmol/L | 49-90 | 72.70 [61.20, 87.00] | 71.10 [61.45, 86.00] | 72.90 [61.20, 87.57] | 0.800 |
| Blood urea nitrogen, mmol/L | 2.8-7.6 | 5.40 [4.49, 6.58] | 5.23 [4.50, 6.17] | 5.47 [4.47, 6.60] | 0.459 |
| Uric acid, μmol/L | 155-357 | 352.90 [284.75, 432.60] | 351.50 [285.40, 415.30] | 353.00 [284.88, 437.63] | 0.992 |
| Serum calcium ion, mmol/L | 2.11-2.52 | 2.26 [2.17, 2.34] | 2.23 [2.13, 2.32] | 2.27 [2.18, 2.35] | *0.009* |
| Serum potassium ion, mmol/L | 3.5-5.3 | 3.90 [3.66, 4.16] | 3.96 [3.70, 4.16] | 3.90 [3.65, 4.16] | 0.497 |
| Serum sodium ion, mmol/L | 137-147 | 140.50 [138.60, 142.10] | 140.50 [138.45, 142.35] | 140.50 [138.60, 142.10] | 0.897 |
| Total cholesterol, mmol/L | <5.18 | 4.51 [3.76, 5.16] | 4.69 [3.99, 5.38] | 4.49 [3.70, 5.12] | *0.028* |
| High density lipoprotein, mmol/L | >1.04 | 1.14 [0.94, 1.37] | 1.10 [0.89, 1.39] | 1.14 [0.95, 1.36] | 0.491 |
| Low density lipoprotein, mmol/L | <3.37 | 2.68 [2.15, 3.22] | 2.87 [2.27, 3.31] | 2.67 [2.12, 3.22] | 0.083 |
| Triglyceride, mmol/L | <1.7 | 1.42 [1.03, 2.00] | 1.35 [0.98, 1.78] | 1.43 [1.03, 2.04] | 0.319 |
| Creatine kinase, U/L | <145 | 86.00 [66.00, 123.00] | 82.00 [62.00, 108.50] | 88.00 [66.00, 126.75] | 0.096 |
| Creatine kinase-MB, U/L | 0-25 | 12.00 [9.00, 17.00] | 12.00 [8.00, 17.00] | 12.00 [9.00, 17.00] | 0.271 |
| Lactate dehydrogenase, U/L | 125-243 | 187.00 [165.00, 220.00] | 181.00 [162.50, 214.50] | 190.00 [166.00, 223.50] | 0.125 |
| Cardiac troponin I, pg/mL | 0-26.2 | 4.00 [2.00, 10.03] | 4.00 [2.10, 10.10] | 4.00 [2.00, 9.90] | 0.799 |
| N-terminal pro-brain natriuretic peptide, pg/mL | <100 | 112.00 [55.75, 357.75] | 112.00 [52.80, 746.00] | 113.00 [57.00, 345.00] | 0.697 |
| **Echocardiography** |  |  |  |  |  |
| Ascending aorta diameter, mm | 20-34 | 33.00 [30.00, 35.00] | 33.00 [30.00, 35.00] | 33.00 [29.08, 35.00] | 0.962 |
| Left atrial diameter, mm | 22-36 | 34.00 [31.00, 37.00] | 35.00 [31.00, 38.00] | 34.00 [31.00, 37.00] | 0.535 |
| Left ventricular diameter, mm | 36-53 | 44.00 [41.00, 47.00] | 44.00 [41.00, 47.00] | 44.00 [41.00, 47.00] | 0.471 |
| Ventricular septal thickness, mm | 6-11 | 11.00 [10.00, 12.00] | 11.00 [10.00, 12.00] | 11.00 [10.00, 12.00] | 0.260 |
| Pulmonary artery diameter, mm | 14-26 | 24.00 [23.00, 26.00] | 25.00 [23.25, 27.00] | 24.00 [23.00, 26.00] | *0.025* |
| LVEF, (%) | 50-75 | 67.00 [61.00, 71.00] | 66.00 [61.75, 71.25] | 67.00 [61.00, 71.00] | 0.890 |
| Severe valve regurgitation, n (%) | Yes | 107 (17.77) | 23 (20.72) | 84 (17.11) | 0.446 |
|  | No | 495 (82.23) | 88 (79.28) | 407 (82.89) |  |
| Severe valve calcification, n (%) | Yes | 94 (15.61) | 21 (18.92) | 73 (14.87) | 0.359 |
|  | No | 508 (84.39) | 90 (81.08) | 418 (85.13) |  |

NA = not available. *P* values ＜0.05 are written in italics.

Values shown are mean ± SD, median (interquartile range [IQR]) or n (%). *P* values were calculated by chi-squared test, Fisher’s exact test, *t* test, or Mann-Whitney *U* test, as appropriate.

Abbreviations: n number, LVEF left ventricular ejection fraction
