# Supplementary material for: The correlation between different antihypertensive treatments and prognosis of cardiovascular disease in hypertensive patients
Source: BMC Cardiovasc Disord. 2023 Jul 22;23:369. doi: 10.1186/s12872-023-03381-x (PMC10363321; doi:10.1186/s12872-023-03381-x)
Supplement: Supplementary file 2 — Additional file 2: Appendix Table 2. Follow-up Characteristics of Hypertension Patients with respect to different antihypertensive treatments. [file 12872_2023_3381_MOESM2_ESM.docx]

**Appendix Table 2. Follow-up Characteristics of Hypertension Patients with respect to different antihypertensive treatments**

| Variables | Normal Range | All patients (n=602) | Irregular (n=111) | Regular (n=491) | *P* value |
| --- | --- | --- | --- | --- | --- |
| Age, years | NA | 69.00 [57.00, 78.00] | 70.00 [56.00, 80.50] | 69.00 [57.00, 78.00] | 0.843 |
| Gender, n (%) | Female | 310 (51.50) | 52 (46.85) | 258 (52.55) | 0.327 |
|  | Male | 292 (48.50) | 59 (53.15) | 233 (47.45) |  |
| Systolic blood pressure, mmHg | 90-139 | 135.00 [123.00, 147.00] | 137.50 [125.00, 147.75] | 134.00 [123.00, 147.00] | 0.217 |
| Diastolic blood pressure, mmHg | 60-89 | 79.00 [70.00, 89.00] | 80.00 [71.00, 90.00] | 78.00 [70.00, 89.00] | 0.164 |
| Heart rate, bpm | 60-100 | 74.00 [68.00, 80.00] | 75.00 [70.00, 80.00] | 74.00 [68.00, 80.00] | 0.563 |
| Diabetes, n (%) | Yes | 158 (26.25) | 20 (18.02) | 138 (28.11) | *0.039* |
|  | No | 444 (73.75) | 91 (81.98) | 353 (71.89) |  |
| Hyperlipidemia, n (%) | Yes | 181 (30.07) | 27 (24.32) | 154 (31.36) | 0.178 |
|  | No | 421 (69.93) | 84 (75.68) | 337 (68.64) |  |
| **Laboratory Findings** |  |  |  |  |  |
| Blood glucose, mmol/L | 3.9-6.1 | 5.39 [4.98, 6.19] | 5.50 [5.12, 6.27] | 5.36 [4.97, 6.13] | 0.129 |
| Serum creatinine, μmol/L | 49-90 | 73.50 [62.32, 93.60] | 73.00 [62.70, 92.70] | 74.00 [62.30, 93.80] | 0.885 |
| Blood urea nitrogen, mmol/L | 2.8-7.6 | 5.75 [4.60, 7.20] | 5.59 [4.61, 6.80] | 5.80 [4.60, 7.29] | 0.560 |
| Uric acid, μmol/L | 155-357 | 364.70 [301.00, 442.10] | 370.70 [306.70, 450.80] | 361.50 [300.53, 441.22] | 0.616 |
| Serum calcium ion, mmol/L | 2.11-2.52 | 2.26 [2.18, 2.35] | 2.26 [2.19, 2.37] | 2.26 [2.18, 2.35] | 0.495 |
| Serum potassium ion, mmol/L | 3.5-5.3 | 3.94 [3.70, 4.16] | 3.92 [3.71, 4.16] | 3.94 [3.70, 4.16] | 0.799 |
| Serum sodium ion, mmol/L | 137-147 | 140.30 [138.20, 142.00] | 139.85 [137.78, 141.10] | 140.40 [138.43, 142.10] | *0.047* |
| Total cholesterol, mmol/L | <5.18 | 4.15 [3.43, 4.87] | 4.31 [3.52, 4.95] | 4.11 [3.42, 4.84] | 0.107 |
| High density lipoprotein, mmol/L | >1.04 | 1.10 [0.93, 1.30] | 1.08 [0.94, 1.35] | 1.11 [0.93, 1.29] | 0.926 |
| Low density lipoprotein, mmol/L | <3.37 | 2.42 [1.76, 3.09] | 2.58 [1.86, 3.25] | 2.39 [1.76, 3.04] | 0.098 |
| Triglyceride, mmol/L | <1.7 | 1.28 [0.93, 1.87] | 1.22 [0.94, 1.79] | 1.29 [0.92, 1.89] | 0.817 |
| Creatine kinase, U/L | <145 | 90.00 [66.25, 130.00] | 83.00 [67.00, 112.00] | 91.00 [66.00, 132.00] | 0.252 |
| Creatine kinase-MB, U/L | 0-25 | 14.00 [10.00, 18.00] | 13.00 [10.00, 18.00] | 14.00 [10.00, 18.00] | 0.400 |
| Lactate dehydrogenase, U/L | 125-243 | 190.00 [165.25, 218.75] | 187.00 [165.00, 213.00] | 190.00 [166.00, 219.00] | 0.823 |
| Cardiac troponin I, pg/mL | 0-26.2 | 4.05 [1.90, 11.53] | 4.50 [2.05, 16.85] | 4.00 [1.90, 10.65] | 0.204 |
| N-terminal pro-brain natriuretic peptide, pg/mL | <100 | 99.85 [50.10, 524.25] | 113.00 [49.05, 2002.50] | 99.40 [50.73, 408.75] | 0.380 |
| **Echocardiography** |  |  |  |  |  |
| Ascending aorta diameter, mm | 20-34 | 33.00 [30.00, 35.00] | 33.00 [30.00, 35.00] | 33.00 [30.00, 35.00] | 0.730 |
| Left atrial diameter, mm | 22-36 | 35.00 [31.00, 40.00] | 35.00 [31.00, 40.00] | 35.00 [31.00, 40.00] | 0.711 |
| Left ventricular diameter, mm | 36-53 | 45.00 [42.00, 48.00] | 44.00 [42.00, 48.00] | 45.00 [42.00, 48.00] | 0.374 |
| Ventricular septal thickness, mm | 6-11 | 11.00 [10.00, 12.00] | 11.00 [10.00, 12.00] | 11.00 [10.00, 12.00] | 0.784 |
| Pulmonary artery diameter, mm | 14-26 | 24.00 [22.00, 26.00] | 24.00 [23.00, 26.00] | 24.00 [22.00, 26.00] | 0.352 |
| LVEF, (%) | 50-75 | 65.00 [59.00, 69.00] | 65.00 [60.00, 70.00] | 64.00 [59.00, 69.00] | 0.430 |
| Severe valve regurgitation, n (%) | Yes | 210 (34.88) | 36 (32.43) | 174 (35.44) | 0.624 |
|  | No | 392 (65.12) | 75 (67.57) | 317 (64.56) |  |
| Severe valve calcification, n (%) | Yes | 140 (23.26) | 29 (26.13) | 111 (22.61) | 0.504 |
|  | No | 462 (76.74) | 82 (73.87) | 380 (77.39) |  |

NA = not available. *P* values ＜0.05 are written in italics.

Values shown are mean ± SD, median (interquartile range [IQR]) or n (%). *P* values were calculated by chi-squared test, Fisher’s exact test, *t* test, or Mann-Whitney *U* test, as appropriate.

Abbreviations: n number, LVEF left ventricular ejection fraction
