# Supplementary material for: The correlation between different antihypertensive treatments and prognosis of cardiovascular disease in hypertensive patients
Source: BMC Cardiovasc Disord. 2023 Jul 22;23:369. doi: 10.1186/s12872-023-03381-x (PMC10363321; doi:10.1186/s12872-023-03381-x)
Supplement: Supplementary file 3 — Additional file 3: Appendix Table 3. Follow-up Characteristics of Hypertension Patients with respect to different antihypertensive treatments. [file 12872_2023_3381_MOESM3_ESM.docx]

**Appendix Table 3. Follow-up Characteristics of Hypertension Patients with respect to different antihypertensive treatments**

| Variables | Normal Range | Regular (n=491) | Single-drug (n=172) | Two-drug (n=213) | Multi-drug (n=106) | *P* value |
| --- | --- | --- | --- | --- | --- | --- |
| Age, years | NA | 69.00 [57.00, 78.00] | 71.00 [60.00, 80.25] | 68.00 [57.00, 76.00] | 66.00 [51.25, 77.00] | *0.022* |
| Gender, n (%) | Female | 258 (52.55) | 105 (61.05) | 105 (49.30) | 48 (45.28) | *0.017* |
|  | Male | 233 (47.45) | 67 (38.95) | 108 (50.70) | 58 (54.72) |  |
| Systolic blood pressure, mmHg | 90-139 | 134.00 [123.00, 147.00] | 132.00 [123.75, 145.00] | 134.00 [123.50, 145.00] | 138.00 [123.00, 149.00] | 0.456 |
| Diastolic blood pressure, mmHg | 60-89 | 78.00 [70.00, 89.00] | 78.00 [70.00, 86.00] | 78.00 [70.00, 89.00] | 80.00 [73.00, 90.00] | 0.229 |
| Heart rate, bpm | 60-100 | 74.00 [68.00, 80.00] | 75.00 [70.00, 80.00] | 72.50 [68.00, 80.00] | 74.00 [68.00, 80.00] | 0.497 |
| Diabetes, n (%) | Yes | 138 (28.11) | 45 (26.16) | 66 (30.99) | 27 (25.47) | 0.458 |
|  | No | 353 (71.89) | 127 (73.84) | 147 (69.01) | 79 (74.53) |  |
| Hyperlipidemia, n (%) | Yes | 154 (31.36) | 57 (33.14) | 58 (27.23) | 39 (36.79) | 0.183 |
|  | No | 337 (68.64) | 115 (66.86) | 155 (72.77) | 67 (63.21) |  |
| **Laboratory Findings** |  |  |  |  |  |  |
| Blood glucose, mmol/L | 3.9-6.1 | 5.36 [4.97, 6.13] | 5.21 [4.84, 5.95] | 5.46 [5.07, 6.23] | 5.40 [4.94, 6.05] | 0.063 |
| Serum creatinine, μmol/L | 49-90 | 74.00 [62.30, 93.80] | 68.15 [58.50, 86.38] | 75.10 [65.00, 93.80] | 79.95 [66.42, 103.72] | *<0.001* |
| Blood urea nitrogen, mmol/L | 2.8-7.6 | 5.80 [4.60, 7.29] | 5.67 [4.42, 7.20] | 5.90 [4.79, 7.14] | 5.76 [4.64, 8.12] | 0.470 |
| Uric acid, μmol/L | 155-357 | 361.50 [300.53, 441.22] | 347.30 [278.72, 425.30] | 362.35 [304.62, 442.30] | 397.20 [328.20, 495.72] | *<0.001* |
| Serum calcium ion, mmol/L | 2.11-2.52 | 2.26 [2.18, 2.35] | 2.24 [2.16, 2.33] | 2.27 [2.20, 2.35] | 2.27 [2.19, 2.35] | 0.230 |
| Serum potassium ion, mmol/L | 3.5-5.3 | 3.94 [3.70, 4.16] | 3.97 [3.74, 4.16] | 3.94 [3.70, 4.16] | 3.90 [3.64, 4.14] | 0.373 |
| Serum sodium ion, mmol/L | 137-147 | 140.40 [138.43, 142.10] | 140.20 [138.10, 142.00] | 140.40 [138.20, 142.00] | 140.90 [139.02, 142.48] | 0.139 |
| Total cholesterol, mmol/L | <5.18 | 4.11 [3.42, 4.84] | 4.19 [3.47, 4.97] | 4.09 [3.42, 4.96] | 3.87 [3.26, 4.48] | *0.043* |
| High density lipoprotein, mmol/L | >1.04 | 1.11 [0.93, 1.29] | 1.13 [0.93, 1.32] | 1.12 [0.93, 1.28] | 1.07 [0.87, 1.25] | 0.159 |
| Low density lipoprotein, mmol/L | <3.37 | 2.39 [1.76, 3.04] | 2.47 [1.84, 3.06] | 2.38 [1.76, 3.16] | 2.24 [1.60, 2.82] | 0.101 |
| Triglyceride, mmol/L | <1.7 | 1.29 [0.92, 1.89] | 1.16 [0.84, 1.82] | 1.30 [0.99, 1.90] | 1.38 [1.04, 1.96] | *0.029* |
| Creatine kinase, U/L | <145 | 91.00 [66.00, 132.00] | 92.00 [64.25, 136.25] | 90.00 [68.00, 115.50] | 102.00 [68.00, 136.00] | 0.539 |
| Creatine kinase-MB, U/L | 0-25 | 14.00 [10.00, 18.00] | 14.00 [10.00, 18.75] | 14.00 [11.00, 19.00] | 13.00 [10.00, 17.00] | 0.615 |
| Lactate dehydrogenase, U/L | 125-243 | 190.00 [166.00, 219.00] | 191.00 [168.00, 223.00] | 189.00 [165.00, 214.75] | 190.00 [166.00, 214.00] | 0.712 |
| Cardiac troponin I, pg/mL | 0-26.2 | 4.00 [1.90, 10.65] | 3.90 [1.90, 11.75] | 4.10 [1.90, 9.00] | 3.90 [1.90, 12.00] | 0.994 |
| N-terminal pro-brain natriuretic peptide, pg/mL | <100 | 99.40 [50.73, 408.75] | 126.00 [53.20, 363.00] | 94.10 [40.50, 311.50] | 159.00 [57.70, 1587.50] | 0.236 |
| **Echocardiography** |  |  |  |  |  |  |
| Ascending aorta diameter, mm | 20-34 | 33.00 [30.00, 35.00] | 33.00 [30.00, 35.00] | 33.00 [30.00, 35.00] | 33.00 [30.00, 35.75] | 0.598 |
| Left atrial diameter, mm | 22-36 | 35.00 [31.00, 40.00] | 34.00 [30.50, 39.00] | 36.00 [32.00, 40.00] | 35.00 [32.00, 39.00] | 0.067 |
| Left ventricular diameter, mm | 36-53 | 45.00 [42.00, 48.00] | 44.00 [41.00, 47.00] | 45.00 [42.00, 49.00] | 46.00 [42.50, 48.00] | *0.034* |
| Ventricular septal thickness, mm | 6-11 | 11.00 [10.00, 12.00] | 10.00 [10.00, 12.00] | 11.00 [10.00, 12.00] | 11.00 [10.00, 12.00] | *0.007* |
| Pulmonary artery diameter, mm | 14-26 | 24.00 [22.00, 26.00] | 24.00 [22.00, 26.00] | 24.00 [22.00, 26.00] | 24.00 [22.25, 26.00] | 0.294 |
| LVEF, (%) | 50-75 | 64.00 [59.00, 69.00] | 65.00 [60.00, 69.00] | 63.95 [59.00, 68.00] | 63.00 [59.00, 70.00] | 0.537 |
| Severe valve regurgitation, n (%) | Yes | 174 (35.44) | 70 (40.70) | 76 (35.68) | 28 (26.42) | 0.053 |
|  | No | 317 (64.56) | 102 (59.30) | 137 (64.32) | 78 (73.58) |  |
| Severe valve calcification, n (%) | Yes | 111 (22.61) | 41 (23.84) | 38 (17.84) | 32 (30.19) | *0.041* |
|  | No | 380 (77.39) | 131 (76.16) | 175 (82.16) | 74 (69.81) |  |

NA = not available. *P* values ＜0.05 are written in italics.

Values shown are mean ± SD, median (interquartile range [IQR]) or n (%). *P* values were calculated by chi-squared test, Fisher’s exact test, t test, or Mann-Whitney U test, as appropriate.

Abbreviations: n number, LVEF left ventricular ejection fraction
