# Supplementary material for: The correlation between different antihypertensive treatments and prognosis of cardiovascular disease in hypertensive patients
Source: BMC Cardiovasc Disord. 2023 Jul 22;23:369. doi: 10.1186/s12872-023-03381-x (PMC10363321; doi:10.1186/s12872-023-03381-x)
Supplement: Supplementary file 4 — Additional file 4: Appendix Figure 1. Kaplan-Meier curves of event-free survival for adverse cardiovascular events over 5 years in patients receiving regular or irregular antihypertensive treatment. Appendix Figure 2. Kaplan-Meier curves of event-free survival for adverse cardiovascular events over 5 years in patients receiving single-drug and two-drug antihypertensive treatment. Appendix Figure 3. Kaplan-Meier curves of event-free survival for adverse cardiovascular events over 5 years in patients receiving ACEis/ARBs + Beta-blockers, ACEis/ARBs + CCBs, Beta-blockers + CCBs and ACEis/ARBs +Diuretics, respectively. Appendix Figure 4. Kaplan-Meier curves of event-free survival for adverse cardiovascular events over 5 years in hypertensive patients on intensive or standard treatment in the two-drug antihypertensive treatment group. Appendix Figure 5. Kaplan-Meier curves of event-free survival for adverse cardiovascular events over 5 years in hypertensive patients on intensive or standard treatment in the single-drug antihypertensive treatment group. [file 12872_2023_3381_MOESM4_ESM.docx]

**
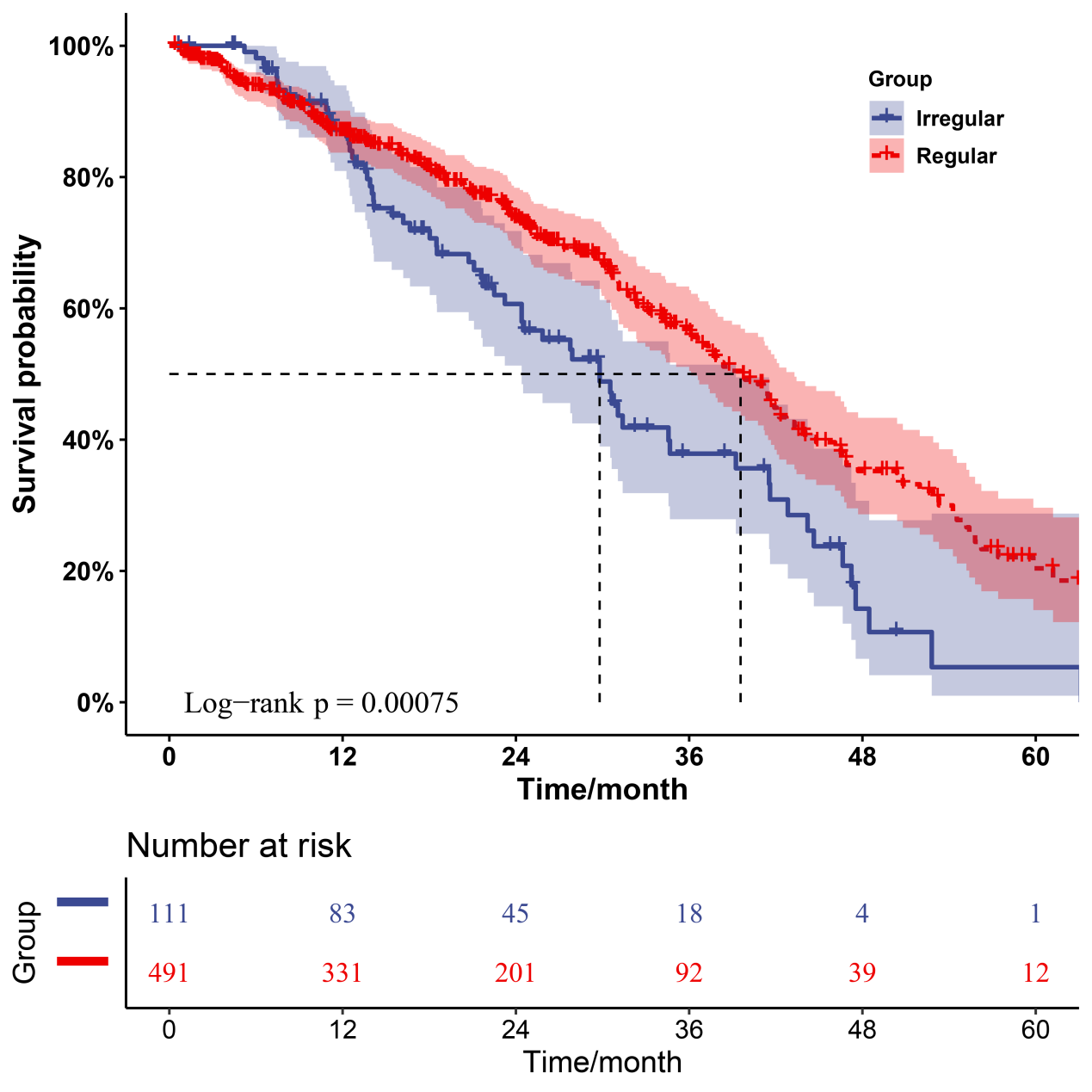
**

**Appendix Figure 1.** Kaplan-Meier curves of event-free survival for adverse cardiovascular events over 5 years in patients receiving regular or irregular antihypertensive treatment.

**
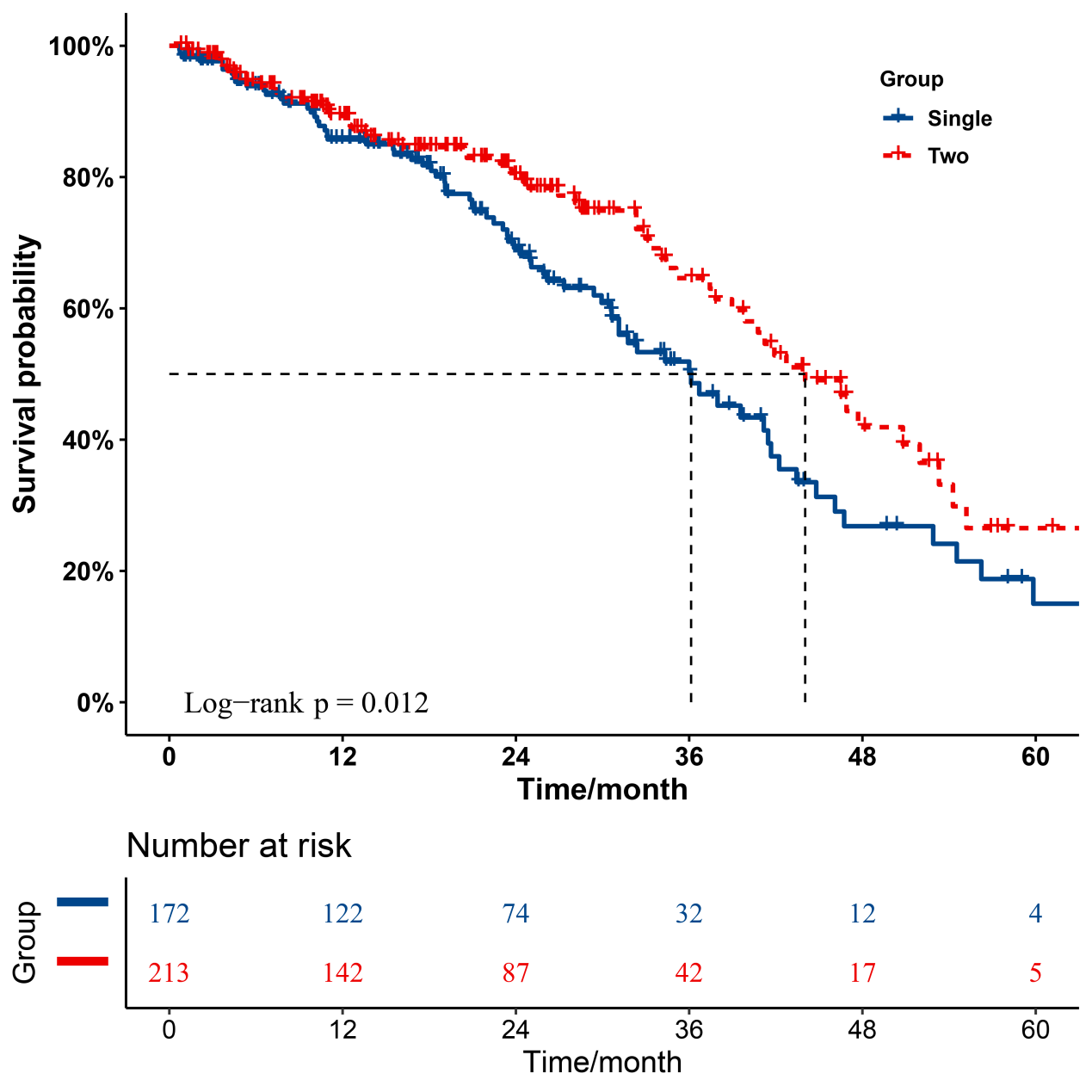
**

**Appendix Figure 2.** Kaplan-Meier curves of event-free survival for adverse cardiovascular events over 5 years in patients receiving single-drug and two-drug antihypertensive treatment.

**
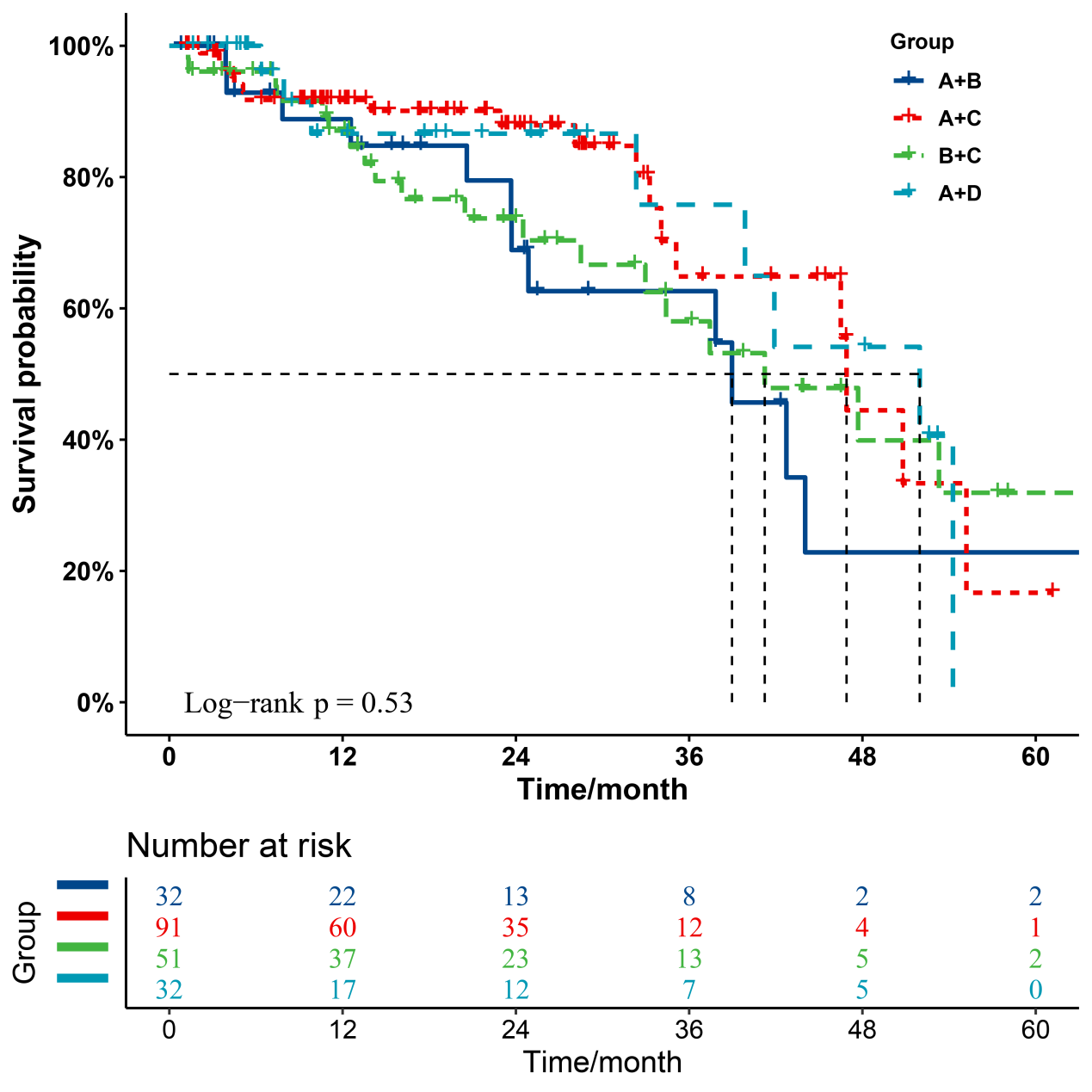
**

**Appendix Figure 3.** Kaplan-Meier curves of event-free survival for adverse cardiovascular events over 5 years in patients receiving ACEis/ARBs + Beta-blockers, ACEis/ARBs + CCBs, Beta-blockers + CCBs and ACEis/ARBs +Diuretics, respectively.

**
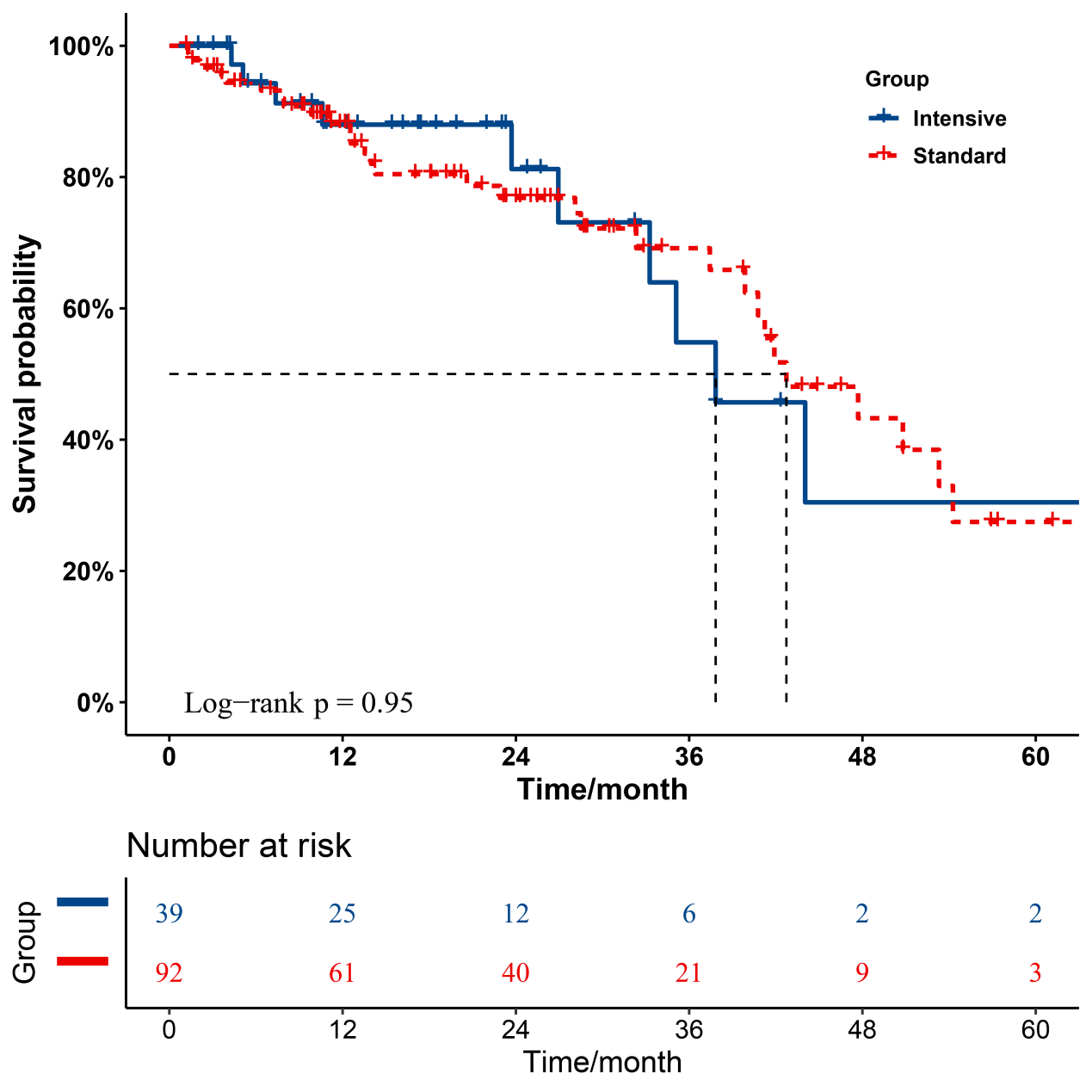
**

**Appendix Figure 4.** Kaplan-Meier curves of event-free survival for adverse cardiovascular events over 5 years in hypertensive patients on intensive or standard treatment in the two-drug antihypertensive treatment group.

**
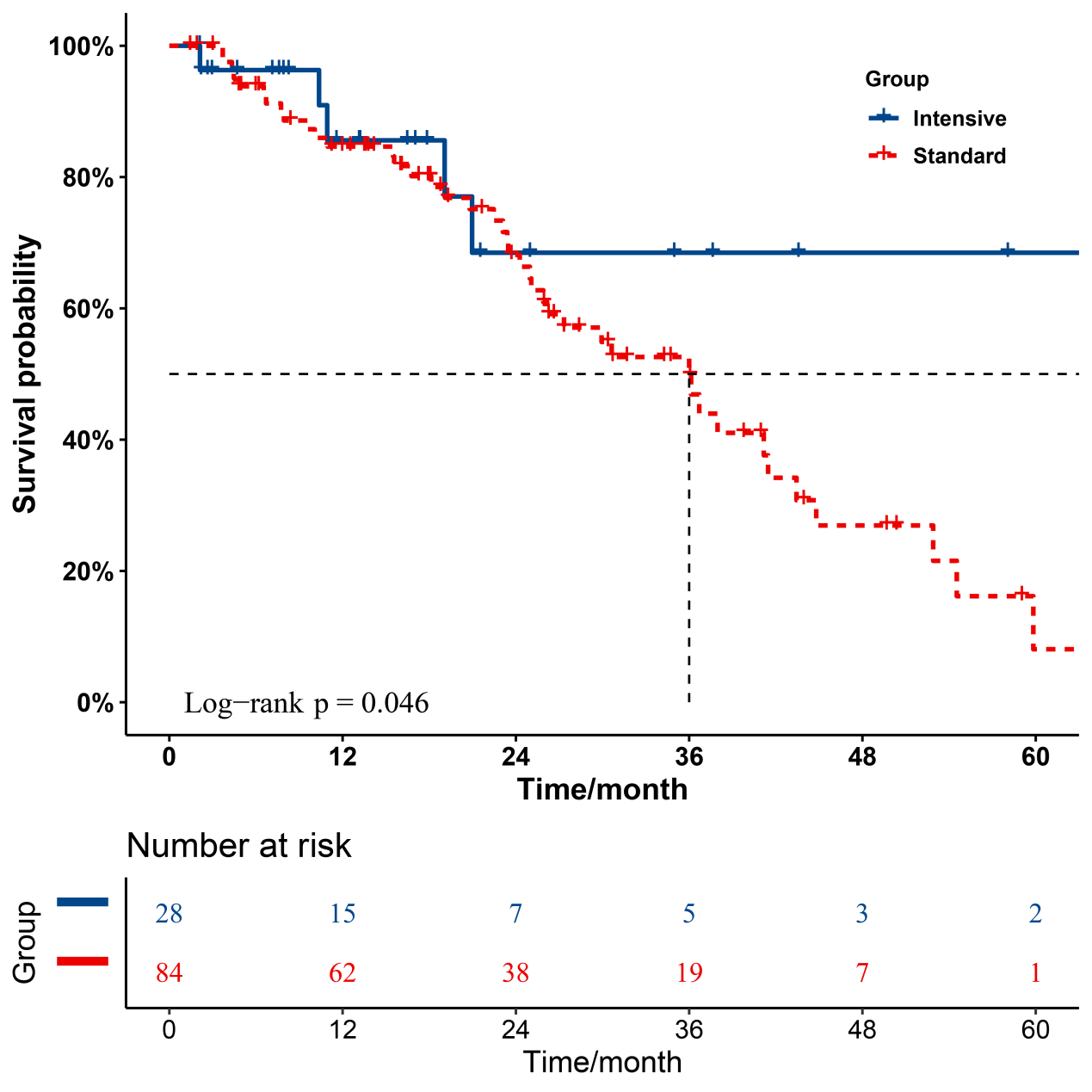
**

**Appendix Figure 5.** Kaplan-Meier curves of event-free survival for adverse cardiovascular events over 5 years in hypertensive patients on intensive or standard treatment in the single-drug antihypertensive treatment group.

**supplementary figure legends**

**Appendix Figure 1.** Kaplan-Meier curves of event-free survival for adverse cardiovascular events over 5 years in patients receiving regular or irregular antihypertensive treatment.

**Appendix Figure 2.** Kaplan-Meier curves of event-free survival for adverse cardiovascular events over 5 years in patients receiving single-drug and two-drug antihypertensive treatment.

**Appendix Figure 3.** Kaplan-Meier curves of event-free survival for adverse cardiovascular events over 5 years in patients receiving ACEis/ARBs + Beta-blockers, ACEis/ARBs + CCBs, Beta-blockers + CCBs and ACEis/ARBs +Diuretics, respectively.

**Appendix Figure 4.** Kaplan-Meier curves of event-free survival for adverse cardiovascular events over 5 years in hypertensive patients on intensive or standard treatment in the two-drug antihypertensive treatment group.

**Appendix Figure 5.** Kaplan-Meier curves of event-free survival for adverse cardiovascular events over 5 years in hypertensive patients on intensive or standard treatment in the single-drug antihypertensive treatment group.
